# Transcript annotation of Chinese sturgeon (*Acipenser sinensis*) using Iso-seq and RNA-seq data

**DOI:** 10.1038/s41597-023-02014-4

**Published:** 2023-02-23

**Authors:** Xiaolin Liao, Libin Zhang, Hua Tian, Bo Yang, Ezhou Wang, Bin Zhu

**Affiliations:** 1 Key Laboratory of Ecological Impacts of Hydraulic-Projects and Restoration of Aquatic Ecosystem, Ministry of Water Resources, Institute of Hydroecology, Ministry of Water Resources and Chinese Academy of Sciences, Wuhan, 430079 China; 2 College of Life Science and Technology, Huazhong University of Science and Technology, Wuhan, 430074 China

**Keywords:** Ichthyology, Genomics

## Abstract

Chinese sturgeon (*Acipenser sinensis*) is a critically endangered fish inhabiting the Yangtze River and Chinese coastal waters. Numerous research projects and conservation efforts have focused on artificial propagation and release to restore this endangered species. However, genomic and full-length transcriptomic sequencing of Chinese sturgeon has rarely been reported. In this study, a total of 10 Chinese sturgeon tissues were used for PacBio Iso-seq and RNA-seq analyses. A total of 19,538 full-length transcripts were obtained with sizes from 51 bp to 7,033 bp. Moreover, cluster analysis of gene families and phylogenetic analysis of 14 species were performed. Furthermore, lncRNAs and coding sequence (CDS) were identified in all Chinese sturgeon tissues. Finally, gene expression profiles and differentially expressed genes (DEGs) were analyzed among 10 tissues in Chinese sturgeon. Taken together, full-length transcripts and the gene expression profile from Chinese sturgeon tissues will provide gene sequences and expression information for future functional genomic study and be very helpful for comprehensive understanding of the genetic mechanism of endangerment in Chinese sturgeon.

## Background & Summary

As an important anadromous fish distributed in Yangtze River and Chinese coastal waters, the Chinese sturgeon (*Acipenser sinensis*) has a long-life span and is one of most primitive vertebrates with a history of over 200 million years^1,2^. Therefore, Chinese sturgeon is a valuable comparative model to study vertebrate evolution, physiology and immunology due to its very long evolutionary history and key phylogenetic position^3,4^. Due to dam construction, overfishing, water pollution and navigation, natural populations of Chinese sturgeon have sharply declined and there has been no spawning in the Yangtze River for eight years since 2013, resulting in extreme danger of going extinct in the wild. The Chinese sturgeon is now a first-class protected animal in China and on the red list of the International Union for the Conservation of Nature (IUCN), and more attention has been paid to artificial propagation and stock release for maintaining the resource^5^. Next-generation sequencing (NGS) technologies have been widely applied in multiple studies, including gene expression pattern analysis, mutation identification, sequence aberrations and alternative splicing event identification^6–10^. The emerging sequencing technology has facilitated transcriptome studies of Chinese sturgeon^5,8,10–13^. However, genome and transcriptome sequencing studies have lagged behind those of other fish species, resulting in limited full-length gene sequence information for Chinese sturgeon. Therefore, transcriptome sequencing could lead to rapid improvements in our understanding of functional genomics in Chinese sturgeon.

Global transcriptome analysis has been widely used for large-scale characterization of functional sequences in non-model species without reference genome information. Transcriptome, especially full-length transcripts data, is the basis and starting point for the study of gene function and structure in non-model organisms without reference genome sequence. However, the transcriptomic and genomic information has not yet been deeply described, resulting in genetic information that is insufficient to explore the genetic endangerment mechanism in Chinese sturgeon.

In this study, we used a total of 10 Chinese sturgeon tissues (including intestine, whisker, kidney, stomach, gill, testis, swim bladder, liver, fin, pancreas) and pooled them for sequencing using PacBio Iso-seq and RNA-seq. A total of 19,538 full-length transcripts were obtained, ranging from 51 bp to 7,033 bp. Bioinformatics analysis showed that 26,731 and 25,095 transcript sequences were annotated to NCBI non-redundant protein sequences (Nr) and Swissprot database, respectively. Moreover, 18,839, 19,971 and 19,260 transcripts were annotated to Kyoto Encyclopedia of Genes and Genomes (KEGG), euKaryotic Ortholog Groups (KOG) and Gene Ontology (GO) databases, respectively. In addition, lncRNAs and coding sequences were identified in all Chinese sturgeon tissues. Taken together, the full-length transcripts and gene expression profiles of Chinese sturgeon will provide gene sequence information for further functional genomic study and be very helpful for a comprehensive understanding of the genetic endangerment mechanism in Chinese sturgeon.

## Methods

### Ethics statement

All fish handling and experimental procedures in this study were approved by the Animal Care and Use Committee of Institute of Hydroecology, Ministry of Water Resources & Chinese Academy of Sciences.

### Sample collection and RNA preparation

A male individual from an artificial reproductive family of Chinese sturgeon was dissected to identify the maturity stage of testis and to sample the tissues for RNA isolation. The sampling individual was 10 years-old with a body length of 207 cm and a weight of 45 kg. In total, 10 tissues (intestine, whisker, kidney, stomach, gill, testis, swim bladder, liver, fin, pancreas) were collected and immediately frozen in liquid nitrogen. The total RNA from each tissue was isolated individually using TRIzol reagent (Invitrogen) according to the manufacturer’s instructions. The total RNA concentration of each tissue was then measured using a NanoDropTM spectrophotometer (Thermo Fisher Scientific). RIN (RNA integrity number) values were calculated using an Agilent 2100 Bioanalyzer (Agilent Technologies).

### PacBio library construction and sequencing

To construct PacBio sequencing libraries, the qualified RNA from the 10 tissues were mixed in equal amounts. Reverse transcription of the RNA mixture was carried out using a SMARTer® PCR cDNA Synthesis Kit. PCR-amplified products were obtained using the BluePippin size selection system (Sage Science, USA) and fragments of length 0.5–6 kb were obtained for library construction. Finally, SMRTbell libraries were prepared using the Pacific Biosciences DNA Template Prep Kit 2.0 and sequenced using polymerase 2.0 and on the PacBio Sequel platform.

### Illumina library construction and sequencing

mRNAs from the 10 tissues of Chinese sturgeons were extracted from total RNA using Dynabeads oligo (dT) (Invitrogen) according to the manufacturer’s instruction (Invitrogen). First- and second-strand cDNA were synthesized using Superscript II reverse transcriptase (Invitrogen) and random hexamer primers. Double-stranded cDNA was fragmented by nebulization and used to generate RNA-seq libraries. The cDNA libraries were sequenced using the Illumina Hiseq X Ten platform to produce 150 bp paired-end reads. The RNA-seq experiments were performed with three biological replicates.

### Pacbio ISO-seq data processing

**Fig. 1:**
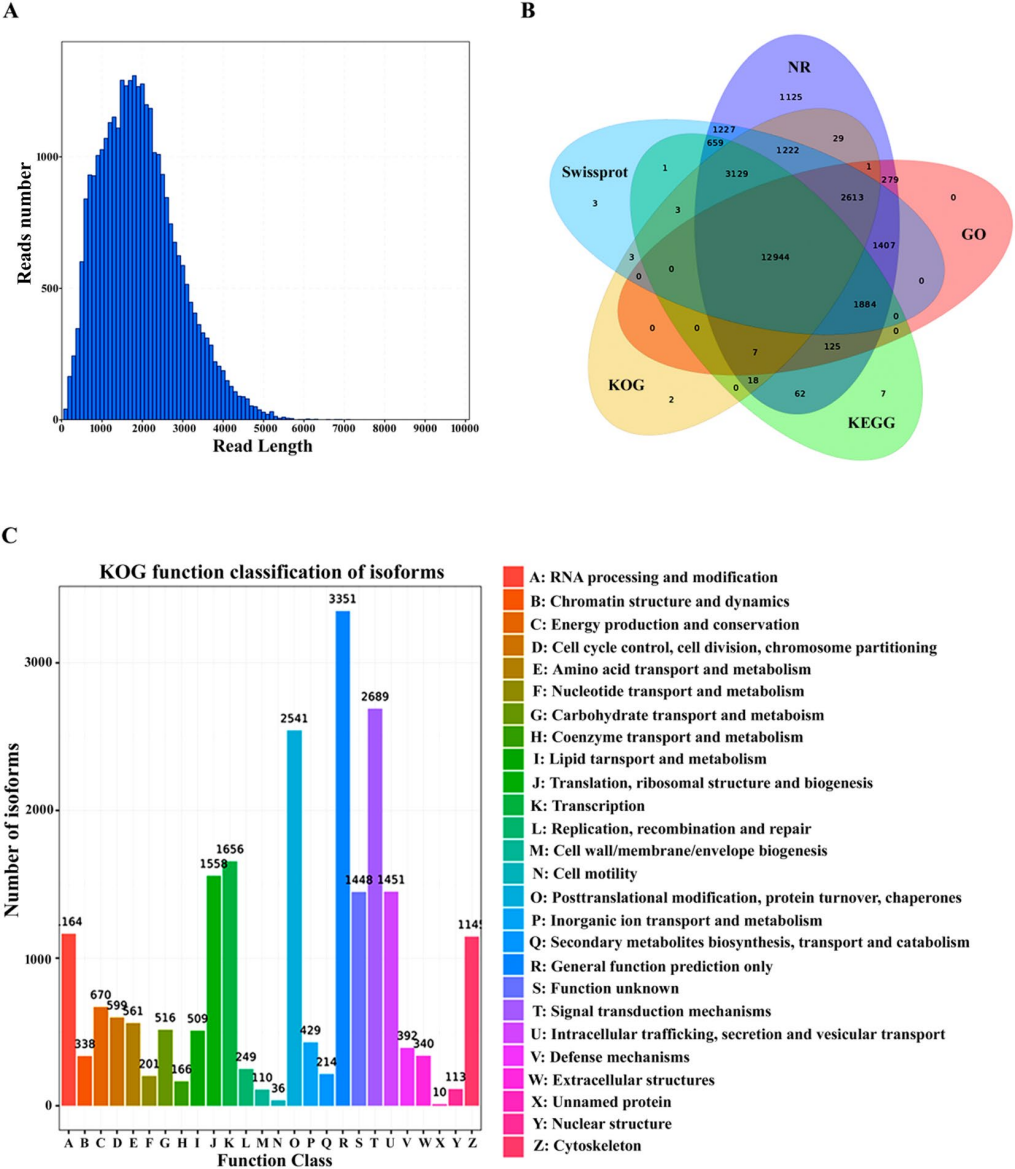
ISO-seq isoforms analysis and annotation in Chinese sturgeon. (**A**). Length distribution of ISO-seq isoforms. (**B**). Venn diagram analysis of ISO-seq isoforms against NR, KOG, Swissprot, GO and KEGG. (**C**). KOG function analysis of ISO-seq isoforms.

As shown in Table 1, Pacbio ISO-seq produced a total of 20,518,995 subreads. Circular consensus sequences (CCSs) of 345,695 reads were generated after self-correction. CCS reads comprised 259,734 FLNC (full-length non-chimeric) reads with an average read length of 1, 689 bp. Furthermore, FLNC reads were used for ICE (Iterative Clustering for Error Correction) analysis to obtain polished transcripts. In addition, polished transcripts were corrected with RNA-seq data using LoRDEC^14^ and removed redundancy, which generated a total of 20,257 high-quality consensus isoforms (accuracy ratio > 99%) with an average length of 1,737 (Fig. 1A). Non-redundant full-length transcript data sets showed high integrity and were used for subsequent analysis.

**Table 1:** Statistic of ISO-sequencing in Chinese sturgeon.

Type	Total bases (bp)	Total reads number	Average length (bp)
Subreads	30.15 G	20,518,995	1,470
CCS	656,725,273	345,695	1,900
FLNC	440,867,755	259,734	1,698
polished transcript	35,190,826	20,257	1,738
Before_correct	35,190,826	20,257	1,738
After_correct	35,185,122	20,257	1,737
Full length transcripts	33,888,937	19,538	1,735

### Function annotation of unigenes

For functional unigene annotation of Chinese sturgeon, the identified unigenes were searched against 7 databases including NR, NT, Swiss-Prot, Pfam, GO, KEGG and COG. BLAST software with an E-value < 1 × 10^−10^ was used in the NT database analysis. The Diamond BLASTX methods with an E-value < 1 × 10^−10^ were analyzed in NR, COG, Swiss-Prot and KEGG annotations. The Hmmscan procedure was used in the Pfam database and GO function categories were performed using the WEGO method.

### Quality control of annotation

Full-length transcripts were annotated with multiple reference databases for further study. First, 16,238 (83.11%), 12,394 (63.44%) and 15,183 (77.71%) transcripts have similar sequences in NCBI non-redundant protein sequences (Nr), KOG and Swissprot, respectively. Moreover, 11,060 (56.61%) and 9,462 (48.43%) transcripts were annotated by KEGG and GO databases, respectively (Fig. 1B). Moreover, ISO isoforms were mapped to KOG database for function classification (Fig. 1C). Furthermore, Blast2GO^15^ was used to assign GO terms and functionally categorize the unigenes of Chinese sturgeon. In order to identify the biological pathways active in Chinese sturgeon, we used the annotated sequences for GO ontology and KEGG pathway analysis. The results of GO term and KEGG pathway analysis were deposited in figshare (10.6084/m9.figshare.22057343.v1). For example, KEGG analysis showed the unigenes were mainly involved in five pathways including Cellular Processes, Environmental Information Processing, Genetic Information Processing, Metabolism and Organismal Systems. In addition, we used transdecoder software to predict and analyze the coding sequences of Chinese sturgeon.

**Fig. 2:**
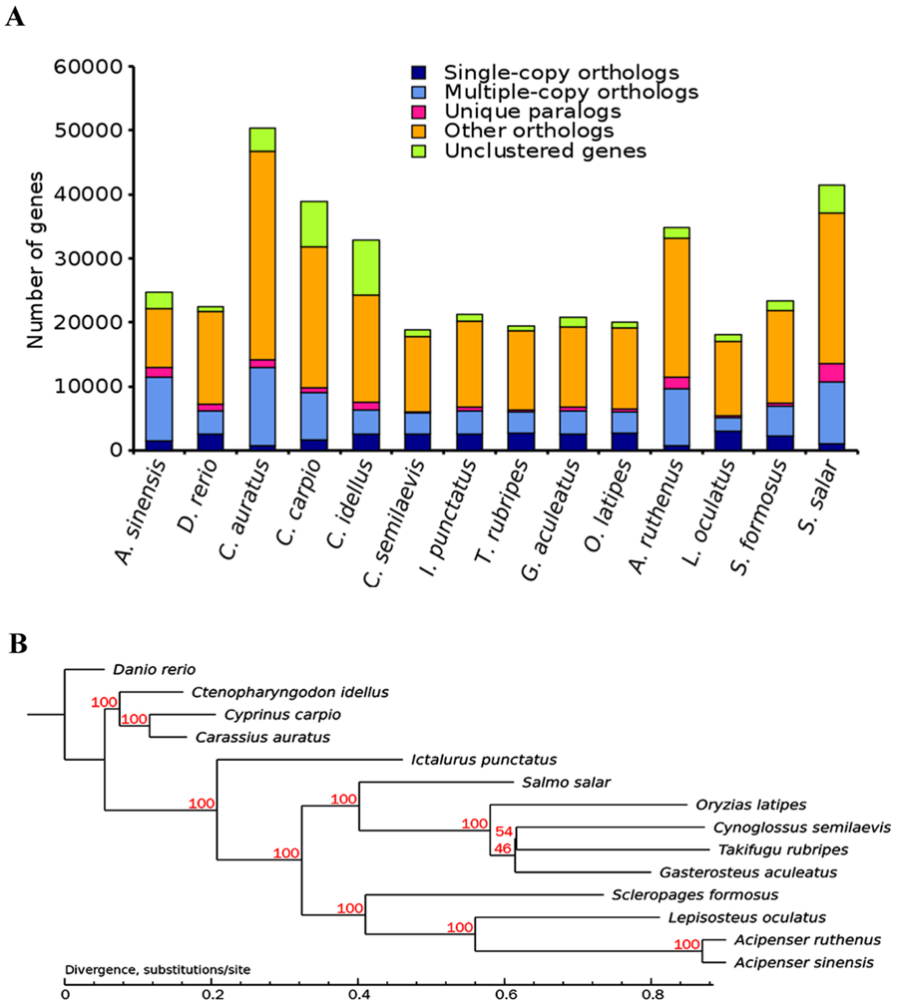
Cluster analysis of gene families and phylogenetic analysis. (**A**). Cluster analysis of gene families of 14 species. (**B**). Phylogenetic analysis of 14 species.

The protein sequences of the 14 species listed later were used for cluster analysis based on sequence similarity (Fig. 2A). All alignments were performed using E-value thresholds of <1e^−5^. As shown in Fig. 2A, a total of 24,759 genes were identified in Chinese sturgeon, including multiple genes homologous with the 14 species in the cluster. RAxML was then used to construct phylogenetic tree using the maximum likelihood method (Fig. 2B). Results indicate that the Chinese sturgeon is closely related to the Sterlet (*A. ruthenus*), a small sturgeon also in the family Acipenseridae. Collectively, the phylogenetic relationship is consistent with the classification and evolutionary status for these species.

**Fig. 3:**
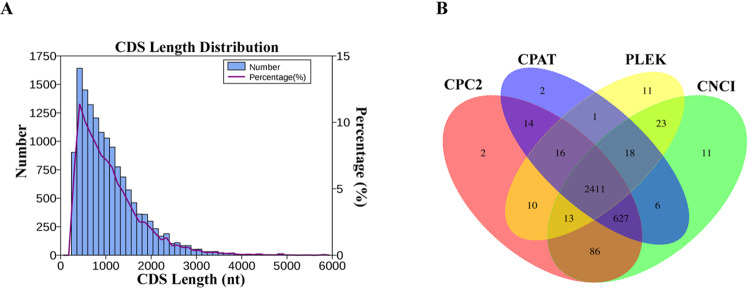
Function prediction of ISO-seq isoforms in Chinese sturgeon. (**A**). CDS prediction of ISO-seq isoforms. (**B**). Venn diagram analysis of predicted lncRNAs of ISO-seq isoforms.

In addition, we also predicted CDS sequences for all full-length transcripts. As shown in Fig. 3A, the lengths of CDS were mainly distributed around 500–1,000 bp in Chinese sturgeon. The longest and shortest lengths of CDS are 6,344 bp and 287 bp, respectively. LncRNAs play important roles in regulating the growth and development of many fish. Here, candidate lncRNAs were predicted using CPC2, CPAT, PLEK and CNCI databases. As shown in Fig. 3B, the Venn diagram analysis of lncRNAs predicted by these four software packages showed that a total of 2411 lncRNAs were identified and deposited in figshare (10.6084/m9.figshare.21995438.v8).

### Predictions of open reading frames (ORFs) and long non-coding RNAs (lncRNAs)

The TransDecoder v2.0.1 package (https://transdecoder.github.io/) was used to predict the ORFs in the transcripts of Chinese sturgeon. Transcripts with complete ORFs and 5′ and 3′ untranslated regions were determined as full-length transcripts. The CNCI^16^, CPC2^17^, CPAT^18^ and PLEK^19^ softwares was used to predict lncRNAs.

### Identification of Differentially Expressed Genes in *Acipenser sinensis*

Correlation analysis and RPKM (Reads Per kb per Million reads) values were used to determine gene expression level in the 10 Chinese sturgeon tissues. The differentially expressed genes (DEGs) were determined with a log-fold expression change (log FC) greater than 2 or less than −2 using a threshold of false discovery rates (FDR < 0.001) and a greater statistically significant value (P < 0.005).

### Cluster analysis of gene families and phylogenetic analysis

The protein sequences of 14 species (*A. sinensis, D. rerio, C. auratus, C. carpio, C. idellus, C. semilaevis, I. punctatus, T. rubripes, G. aculeatus, O. latipes*, *A. ruthenus, L. oculatus, S. formosus* and *S. salar*) were used for cluster analysis based on sequence similarity^20^. The overlapped single copy orthologous genes were filtered out and only genes with amino acid lengths ≥ 100 were retained and used to carry out multiple sequence alignment using MUSCLE^21^. The results of multiple sequence alignment were further combined and transformed into super gene alignment in phylip format. Finally, RAxML^22^ was used to construct a phylogenetic tree by the maximum likelihood method.

## Data Records

All RNA-seq raw reads of Chinese sturgeon were deposited in the Sequence Read Archive (SRA) of the National Center for Biotechnology Information under accession number SRR15886373, SRR15886372, SRR15886371, SRR15886370, SRR15886369, SRR15886368, SRR15886367, SRR15886366, SRR15886365, SRR15908259, SRR15908258, SRR15908257, SRR15908256, SRR15908255, SRR15908254, SRR15908253, SRR15908252, SRR15908251, SRR15908250, SRR15908249, SRR15908248, SRR15908247, SRR15908246, SRR15908245, SRR15908244, SRR15908243, SRR15908242, SRR15908241, SRR15908240 and SRR15908239^23–52^. All raw full-length Iso-seq reads of A. *sinensis* were deposited in the Sequence Read Archive (SRA) of the National Center for Biotechnology Information under accession number SRR15884198^53^. Moreover, the results of GO term and KEGG pathway analysis for the annotated sequences of Chinese sturgeon were deposited in figshare^54^. Furthermore, the LncRNA prediction results of Chinese sturgeon were deposited in figshare^55^.

## Technical Validation

**Fig. 4:**
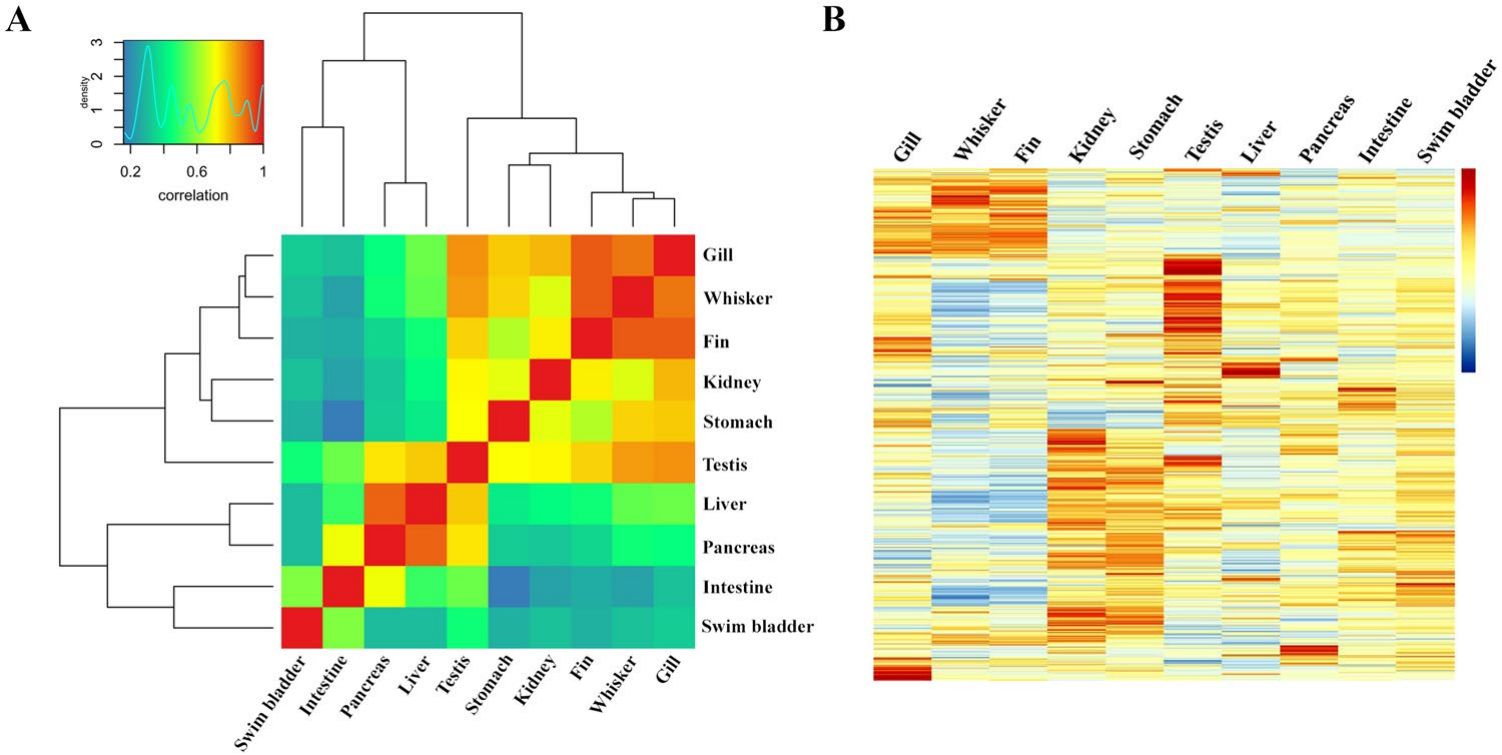
Gene expression profile in 10 tissues (gill, whisker, fin, kidney, stomach, testis, liver, pancreas, intestine, swim bladder) in Chinese sturgeon. (**A**). The correlation analysis of the clean reads of 10 tissues. (**B**). Heatmap analysis of the differentially expressed mRNAs in 10 tissues of Chinese sturgeon.

To investigate global mRNA expression patterns in Chinese sturgeon, a total of 10 tissue samples (gill, whisker, fin, kidney, stomach, testis, liver, pancreas, intestine, swim bladder) of Chinese sturgeon (each run in triplicate) were sequenced using the Illumina Hi-Seq. 2000 platform. FastQC was used to assess the quality of clean reads by Illumina RNA-seq. The average effective rate (Clean reads/Raw reads) was 94.7%. The average Q20 and Q30 were 96.1% and 88.0%, respectively. Moreover, the GC contents of the Chinese sturgeon tissue samples were normally distributed, indicating the sequencing data was not contaminated. As there is no available reference genome information for Chinese sturgeon, the clean reads of the 10 tissue samples were mapped to the isoforms of Chinese sturgeon generated from Pacbio ISO-seq. The Correlation analysis was carried out (Figure 4A) and the gene expression profile were analyzed among 10 Chinese sturgeon tissues (Figure 4B).

## Data Availability

All software used in this study are in public and their parameters are clearly described in Methods. If no detail parameters were mentioned for the software, default parameters were used as suggested by developer.
